# Exploring the Structural Basis of Cryptic Pocket Formation Driven by Extensive Protein Conformational Changes in Drug Targets

**DOI:** 10.1021/acs.jctc.5c02016

**Published:** 2026-03-04

**Authors:** Martijn P. Bemelmans, Alberto Borsatto, Simone Marsili, Francesco L. Gervasio, Vineet Pande

**Affiliations:** 1 Computer-Aided Drug Design, In Silico Discovery, Therapeutics Discovery, Johnson & Johnson Innovative Medicine, Turnhoutseweg 30, Beerse 2340, Belgium; 2 School of Pharmaceutical Sciences, University of Geneva, Rue Michel Servet 1, Geneva 1206, Switzerland; 3 Swiss Bioinformatics Institute, University of Geneva, Geneva 1206, Switzerland; 4 Computer-Aided Drug Design, In Silico Discovery, Therapeutics Discovery, Johnson & Johnson Innovative Medicine, C. Río Jarama, 75, Toledo 45007, Spain; 5 Chemistry Department, University College London (UCL), London WC1E 6BT, U.K.

## Abstract

Allosteric pockets that typically only emerge in the presence of a binder, known as cryptic pockets, can provide an avenue for drug discovery in challenging pharmaceutical targets. However, protein conformations exposing cryptic pockets are generally short-lived and can require significant structural rearrangements that complicate their discovery in experiment and simulation. Here, we investigate the structural basis of cryptic pocket formation in drug targets characterized by extensive dynamics using simulation-based methods. We find that functional protein segments can be anchored by local intramolecular contacts and that disrupting these interactions drives undirected large conformational changes to form cryptic pockets in PRMT5, PRMT6, SMARCA2, Abl1, and PI3Kα. Perturbing the contact networks with benzene probes, elevated temperature, or scaled protein–water interactions could not facilitate these structural dynamics here, indicating that complex mechanisms involving high-energy barriers are necessary to form ligandable cryptic pockets. Based on these limitations, a new computational approach was developed to guide conformational sampling by local interactions surrounding functional protein segments, termed “SLICE” (sampling by local interaction-guided conformational exploration). Across multiple pharmaceutically relevant proteins, our simulations aid in understanding and rapidly exploring the large-scale structural plasticity governed by the local protein environment around functional segments that can be leveraged for drug discovery.

## Graphical abstract

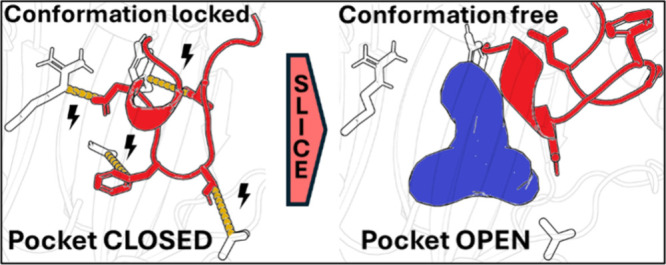

## Introduction

Conformational flexibility is a feature of proteins that can be fundamental to their biological function and molecular recognition.^1^ Accordingly, it is often explored in drug discovery, and structural biology combined with computational efforts aimed at exploring conformational ensembles are applied to inform molecular design.^2^ The significance of considering structural plasticity in drug discovery was recently illustrated by Fajer et al.,^3^ and Saar et al.,^4^ who respectively reported the use of multiple protein conformations to understand differences in potencies of Abl1 inhibitors or SARS-CoV-2 M^Pro^ binder affinities. Moreover, in some cases significant structural changes are required for ligandable binding sites to form, known as cryptic pockets, which can evade detection by high-resolution structure determination of unliganded proteins.^5^ Indeed, early experimentally resolved structures of some pharmaceutical targets lacked druggable pockets, including MYC,^6^ p53,^7^ and Ebola VP35.^8^ These proteins have since been targeted by molecules binding to pockets that emerged upon structural changes (Figure 1A), highlighting the therapeutic potential of cryptic pockets.^6–8^ However, it is not always evident what drives their formation, especially when extensive dynamics are involved. This complicates the ability to rationally obtain informative structural ensembles that can be used to understand protein–ligand binding early in drug discovery campaigns.^9^

**Figure 1:**
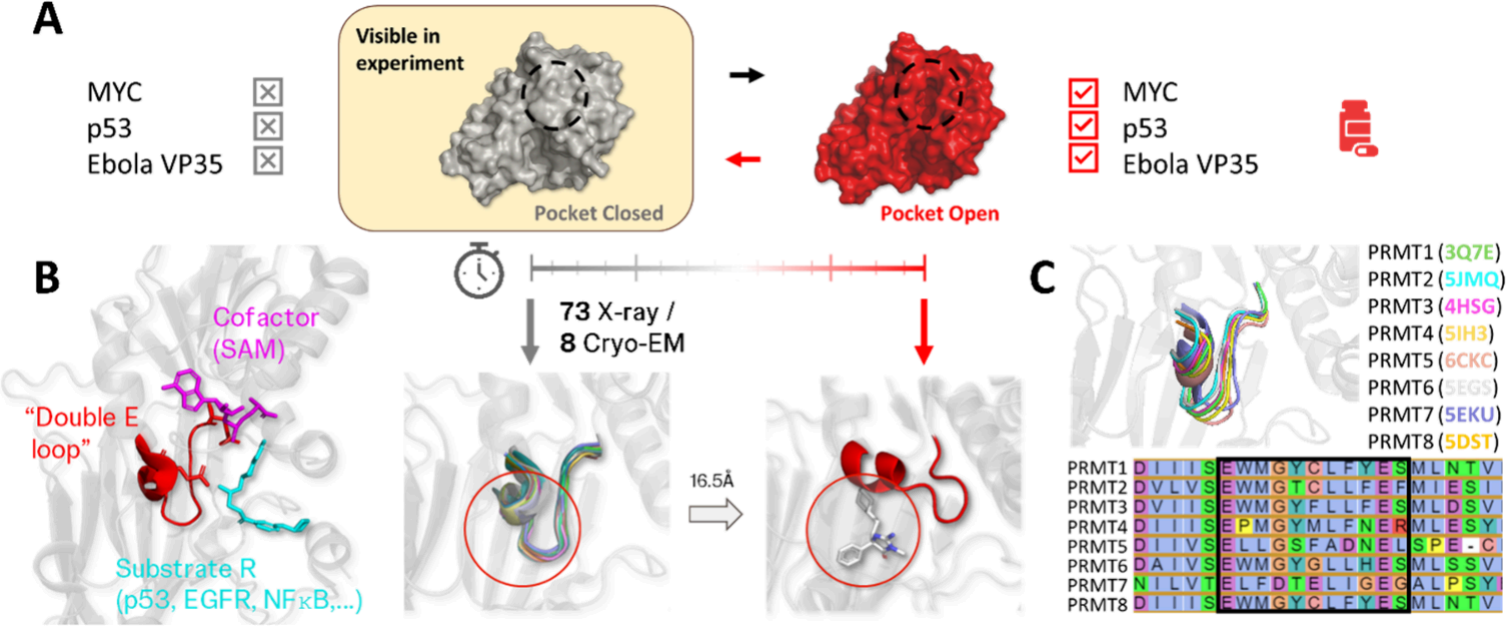
Cryptic pockets and the case of protein arginine methyltransferase 5. (A) Illustrates how early experimentally resolved structures of unliganded drug targets lacked satisfactory pockets for drug discovery but dynamic structural changes revealed cryptic pockets enabling ligand binding. (B) Shows PRMT5′s functional “double E” loop (red), cofactor SAM (magenta) and an example substrate arginine (cyan). An overlay of experimentally resolved structures with the EE loop in its default position is also shown, along with an experimentally resolved state (PDB ID 6UXY) with the exposed cryptic pocket (circled in red). (C) Shows a structure and sequence alignment (EE loop boxed in black, Clustal2 coloring) of PRMT5 with the other PRMT family members, highlighting EE loop conservation.

It is now understood that cryptic pockets can form through a variety of protein motions, ranging from side chain rotations to secondary structure displacement and domain shifts.^5^ Mechanistic investigation by molecular dynamics (MD) simulations revealed that these motions occur by an interplay of conformational selection and induced-fit effects, with relative contributions varying based on the protein context. For instance, in Lymphocyte Function-associated Antigen 1 (LFA-1) conformational selection is dominant and the structural changes involved in cryptic pocket formation can be captured in the accessible ensemble with standard MD.^10^ For KRAS^G12D^, conversely, the induced-fit component dominates and molecular probes in a mixed-solvent MD (MSMD) protocol are needed to incite dynamics for pocket formation.^11^ Simulations with “Sampling Water Interfaces through Scaled Hamiltonians” (SWISH) showed that buried protein regions require a more equally mixed mechanism. In TEM-1 β-lactamase, for example, backbone motions in the conformational ensemble are necessary before probes can access and further induce the cryptic pocket.^12^ When more complex dynamics with high energy barriers are involved, however, including both mechanistic effects by SWISH and probes can remain insufficient for cryptic pocket formation at practical simulation times and require further developments like SWISH-X.^13^ In this context, pockets whose exposure involves functional loops, which can be characterized by extensive allosteric conformational transitions and high energy barriers, pose a relevant drug discovery scenario that cannot be anticipated.^14^

A recent example of this is Protein Arginine Methyltransferase 5 (PRMT5); an epigenetic enzyme that transfers methyl groups from cofactor S-adenosyl methionine (SAM) to histone and nonhistone substrates, including EGFR, p53, and NF-kB^15^ (Figure 1B). This post-translational modification, which makes PRMT5 a regulator of cell proliferation, cell cycle progression, and cell death, is carried out by an 11-residue loop containing two glutamate residues;^15,16^ hereafter the EE loop. According to data in the Protein Databank (PDB)^17^ from April 2025, PRMT5 has been structurally solved at least 83 times, including 75 times by X-ray crystallography and 8 times by cryo-EM. These structures show the EE loop to adopt a near identical conformation in each experiment (Figure 1B). That is except for twice, when different high-throughput screening hits from Merck & Co. were cocrystallized with PRMT5, revealing the EE loop to be displaced by a reported 16.5Å to open a cryptic pocket accommodating the allosteric small molecule inhibitors^18^ (Figure 1B, PDB ID 6UXX and 6UXY). This was a unique finding given the previously reported stability of the EE loop in PRMT5 and the rest of the PRMT family, where the EE loop is relatively conserved both in structure and sequence (Figure 1C).

Although PRMT5′s cryptic pocket has now been discovered, we wondered if simulation approaches could have helped to consider its formation at an early drug discovery stage where ligand information is not available. Recent efforts from the D.E. Shaw research group (100 μs) and Folding@home project (0.1s)^19,20^ have shown that rare protein states can be uncovered through prolonged MD simulations, but the time and resources required are not always available. In this context, adaptive-bias methods like metadynamics^21^ have been established as an effective approach to drive extensive conformational dynamics. They operate by adding an external bias to the system’s potential energy along selected degrees of freedom, known as collective variables (CVs), to overcome energy barriers and broaden the accessible conformational ensemble. Whereas metadynamics builds a bias to gradually discourage revisiting well-explored coordinates, the recent “On-the-fly Probability Enhanced Sampling” (OPES) protocol^22,23^ instead iteratively estimates a probability distribution to define a dynamic bias that directly encourages visiting less-explored coordinates. This reinterpretation of metadynamics should thus enable greater conformational exploration at short simulation times. However, the value of OPES-based methods in cryptic pocket identification remains to be substantiated.

In this setting, our goal was to develop a general method that is suitable for drug discovery and does not rely on *a priori* information or prolonged simulations, while gaining insights in what drives extensive structural changes to form cryptic pockets. We mimic a realistic early discovery scenario by assuming our only information is a single X-ray structure of PRMT5 with the default EE loop conformation and the established fact that this loop is essential for enzymatic function. Information regarding the location of the cryptic pocket, the conformation revealing it, and the allosteric binders is deliberately excluded. To assess robustness in broader protein classes, we also investigate cryptic pocket formation in other drug targets known to undergo significant structural changes.

## Results

### The EE Loop in PRMT5 Is Structurally Locked by Resilient Local Protein Contacts

We started investigating the ability of established MD protocols to sample EE loop dynamics. In our setup, standard MD left the EE loop relatively static even after 1 μs, with an average root mean squared deviation (RMSD) over three replicas of 1.5 ± 0.1Å compared to 2.6 ± 0.2Å for the rest of the protein (Supporting Figure S1). Perturbing the EE loop with REST2-MD^24,25^ (1 μs) and MSMD (500 ns), which respectively attempt to drive protein opening motions by raising the effective temperature or including probes (see methods section), also did not enhance EE loop dynamics (average RMSD_REST2‑MD_ = 1.7 ± 0.2Å, average RMSD_MSMD_ = 1.4 ± 0.2Å). SWISH, which uses benzene probes along with scaled protein–water interaction parameters even distorts parts of the protein structure (Supporting Figure S2A) while only marginally affecting EE loop motion (average RMSD = 1.6 ± 0.4). To localize bias to the EE loop without steering to a predetermined conformation, metadynamics (200 ns) was next employed using the Cartesian coordinates of the center of mass (COM) of the EE loop as three separate CVs. While this slightly enhanced EE loop displacement compared to other methods (average RMSD 2.4 ± 0.6), motions remained comparable to the rest of the protein (average RMSD 2.8 ± 0.5), and one replica even displayed partial structural distortion (Supporting Figure S2B). Together, this observed resilience to displacement indicates that PRMT5′s EE loop is structurally rigidified by local protein interactions that are not easily disrupted.

### Guiding Conformational Sampling by the Local Protein Contacts of the EE Loop Rapidly Yields Extensive Structural Changes That Form a Ligandable Cryptic Pocket in PRMT5

To perturb the local EE loop environment more robustly at practical simulation times, the COM_X,Y,Z_ CVs were subjected to an OPES bias. This produced considerably more EE loop dynamics (RMSD 9.0 ± 1.0Å) than all other methods and did so in the shortest sampling time (100 ns). Inspired by these initial observations, we sought to develop an enhanced sampling approach that can mimic the strong induced-fit effects of ligands and disrupt the local contacts between the EE loop and its surroundings. This led to the OPES-based method SLICE (Sampling by Local Interaction-guided Conformational Exploration) (Figure 2), which guides conformational dynamics by biasing (OPES Explore^23^) the relative distances between the EE loop and its close contacts. Herein, close contacts are defined as residue pairs where the center of mass (COM) of a residue side chain in the EE loop lies within 4.5Å of a residue side chain COM outside the EE loop. In PRMT5, SLICE resulted in simultaneous biasing of four close contact distances, being L437-Y468, S439–V503, F440–S470, and D442-R604 (Figure 2), which rapidly led to structural dynamics beyond the extent observed in the established simulation protocols.

**Figure 2:**
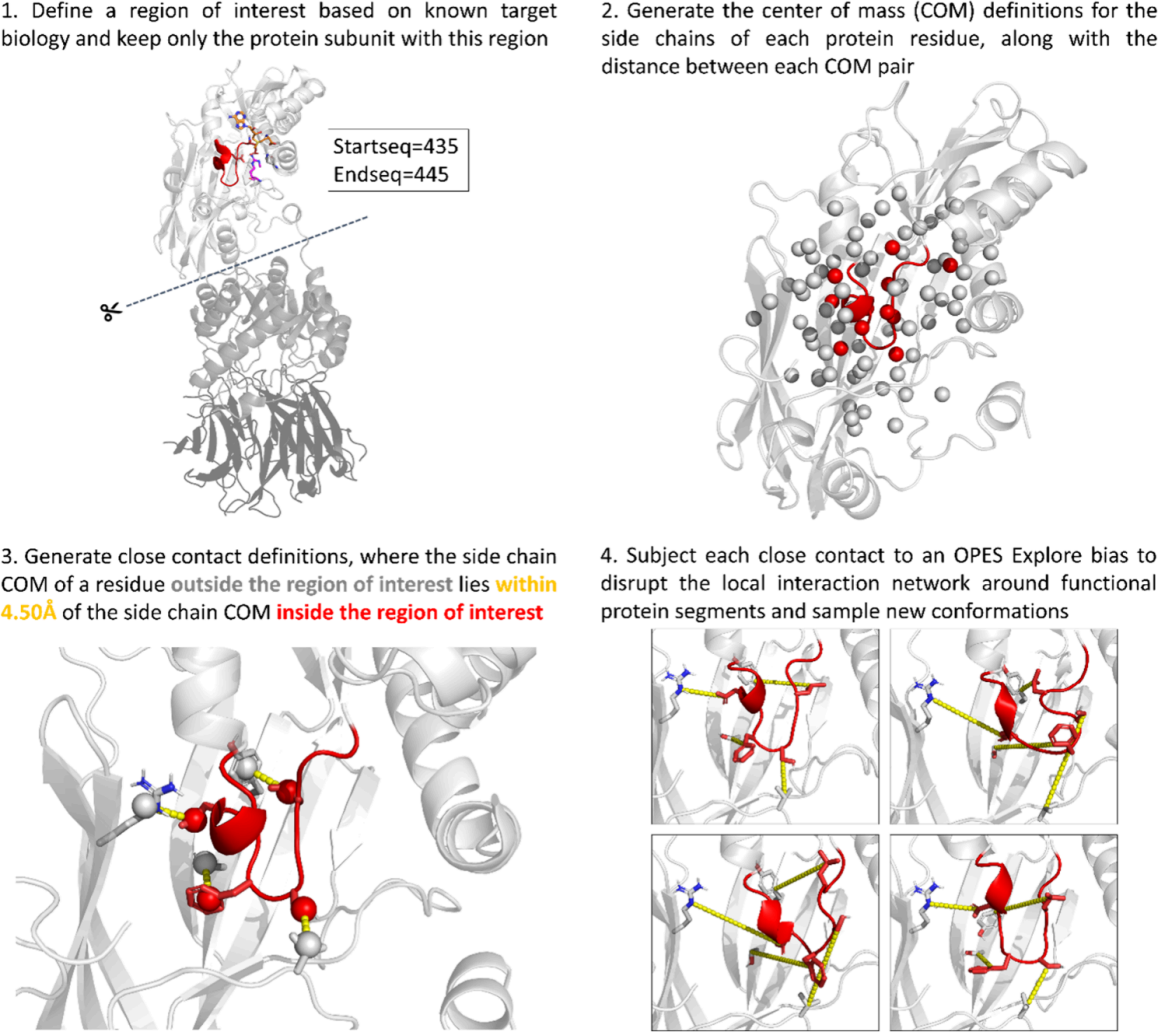
SLICE sampling method based on close contact CVs and OPES Explore biases. Here, the key steps involved in the developed sampling method SLICE are depicted. Knowledge of the target biology is used first to manually set the start and end sequence position to define a region of interest, upon which close contacts are automatically defined and disrupted through OPES Explore biases in a molecular dynamics simulation. PRMT5 is shown as an example here, with panel 4 illustrating various sampled conformations of the EE loop and its close contacts.

From a drug discovery perspective, these structural rearrangements are only valuable if they expose a pocket that can be occupied by a ligand to lock the EE loop in a new state. To detect the presence of such pockets, Schödinger’s SiteMap^26^ was applied on the triplicate SLICE trajectories, producing numerous candidate pockets (Figure 3A). Notably, these include known binding sites, such as the cofactor and substrate site, as well as superficial binding areas. Druggable pockets with a strong cryptic nature were then selected by filtering out sites that already emerge in 100 ns of standard MD (Figure 3B) or are poorly druggable according to SiteScore and DScore.^26^ In PRMT5, this highlighted a single pocket region, which fully accommodates the allosteric ligand coordinates from crystal structures (Figure 3B, PDB ID 6UXX, 6UXY). This shows that the extensive conformational changes underlying cryptic pocket formation in PRMT5 can be unlocked by disrupting the local contact network of the EE loop without requiring prior information on the pocket, its binders, or a protein state revealing it.

**Figure 3:**
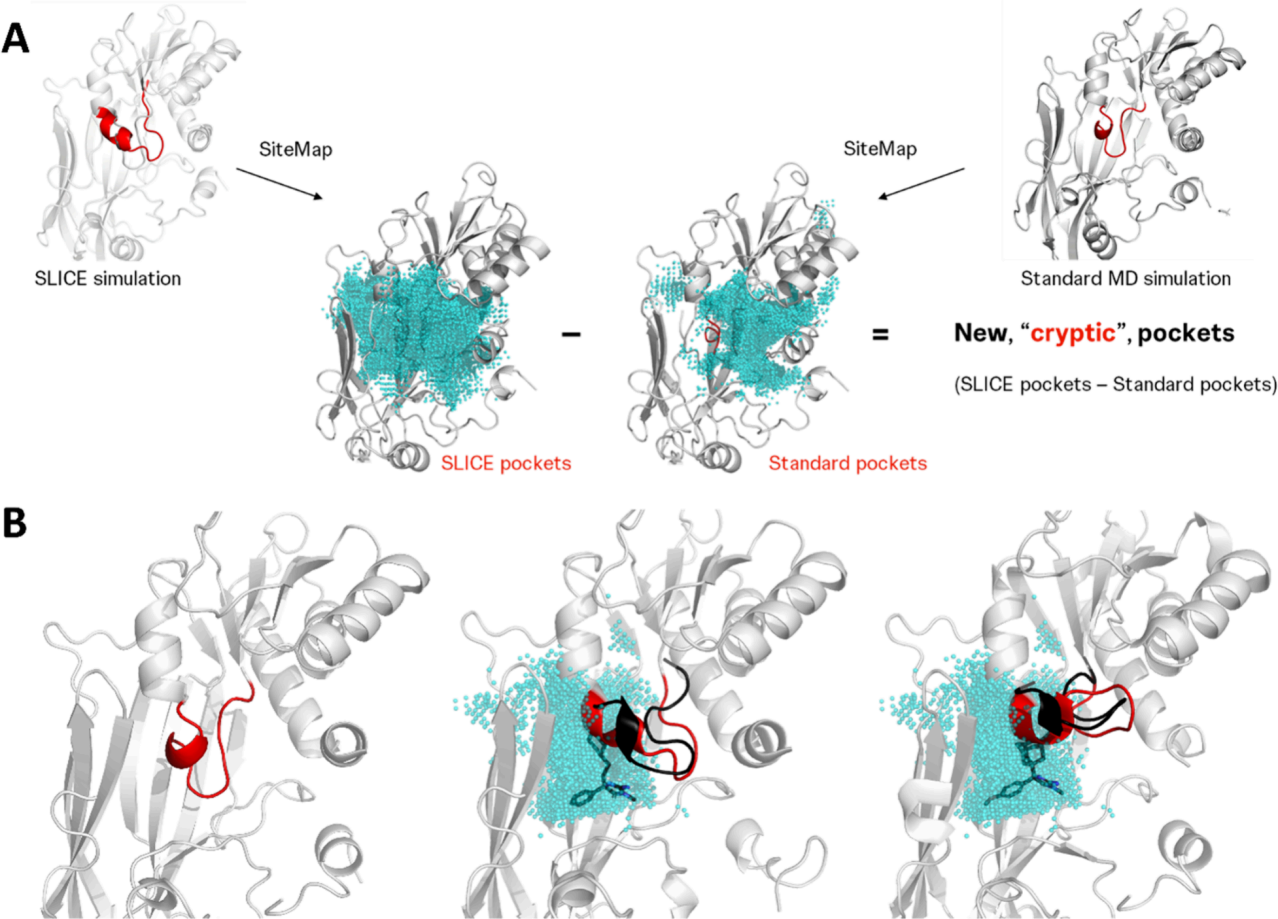
Pocket analysis and results for PRMT5. (A) Depicts our approach to define ligandable cryptic pockets, where binding regions identified by SiteMap (cyan spheres, SiteScore > 1.0, DScore > 1.0) in the SLICE simulations are only retained if there is no significant (>20%) overlap with binding regions identified by SiteMap in the standard MD simulations. (B) Shows the resulting identified ligandable cryptic pocket for PRMT5 (cyan spheres), along with the crystallized (red, PDB ID 7KIC, 6UXY, and 6UXX) and example simulated (black) EE loop conformations. For reference, the cocrystallized allosteric ligands (black sticks) are also shown but they were not part of the simulations or pocket definitions.

### Disrupting the Local Contact Network of Functional Segments in Other Pharmaceutical Targets Similarly Drives Extensive Dynamics to Form Ligandable Cryptic Pockets

Based on the results obtained for PRMT5, we wondered if cryptic pocket formation for other pharmaceutically relevant proteins could also be driven by perturbing the local contacts of functional segments. To this end, SLICE was tested on four additional systems characterized by extensive conformational changes to form cryptic pockets: PRMT6, Abl1 kinase, SMARCA2, and PI3Kα. PRMT6 bears a similar cryptic pocket to PRMT5 that also emerges from EE loop dynamics, though less pronounced. Guiding conformational sampling by biasing local contacts around the EE loop also drove cryptic pocket formation in PRMT6 (Figure 4A), as noted by its overlapping coordinates with a cocrystallized allosteric ligand (PDB ID 6W6D). Beyond methyltransferases, the Abl1 kinase is a classic example displaying extensive conformational dynamics that can be challenging to simulate. Starting from an active “DFG-in” state, a near full flip of the activation loop was sampled by SLICE to resemble a “DFG-out” state and enable identification of a pocket growth vector that was initially covered by the DFG motif (Figure 4B). While this was already sufficient to help identify the cryptic subpocket, additional replicas notably also sampled a full and spontaneous DFG flip (Supporting Figure S3).

**Figure 4:**
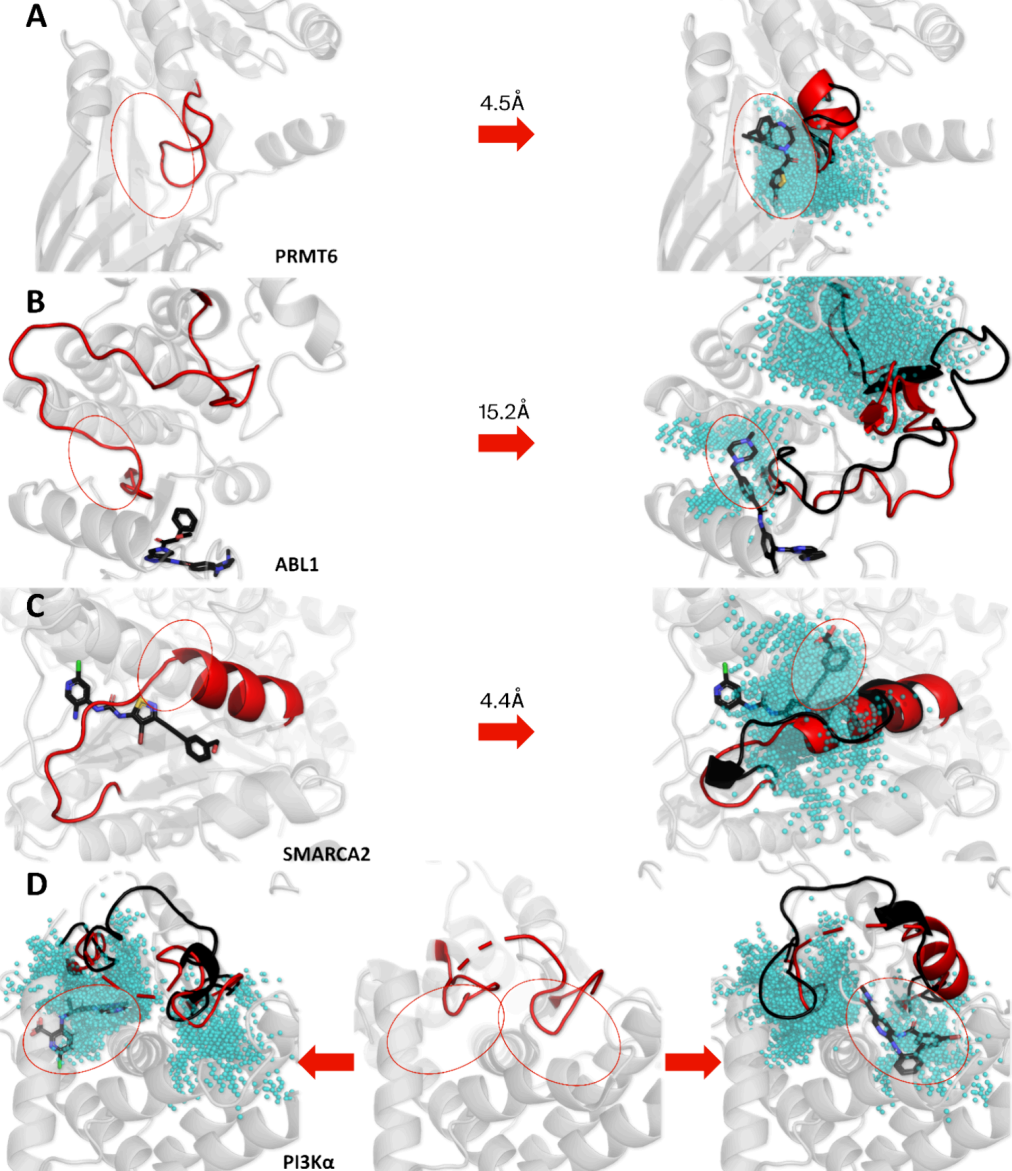
SLICE cryptic pocket detection results in other pharmaceutical targets. In all cases, the detected cryptic pockets are shown as cyan spheres, the structural elements involved in the main dynamic shift to form the cryptic pockets are shown in red, and cryptic ligands along with an example state from simulation are shown in black. For (A–C), an *apo* starting structure is shown on the left, while a crystal structure of a *holo* form with the cryptic pocket exposed is shown on the right. For (D), an *apo* crystal is shown in the middle, while the two crystallized cryptic states are shown on either side. The backbone RMSD of the region displayed in red is also shown for each structure, except for PI3Kα, as it contains missing residues in this region.

In all these cases, the region of interest could be defined by selecting the protein segment involved in the enzymatic function of the target, designating the EE loop in PRMT5 and PRMT6, and the activation loop in Abl1. To assess if the method and structural insights are more broadly applicable, SMARCA2 was investigated next. This protein has two exemplary PDB structures showing one ligand series that can grow in two different vectors, depending on dynamic shifts of a proximal α helix and loop segment (PDB ID 6EG2 and 6EG3). Here, SLICE enabled the identification of a single pocket area covering both directions available for ligand growth (Figure 4C). As a final example, PI3Kα has a loop in its catalytic subunit that lacks electron density in an *apo* crystal structure (PDB 8TS7), indicating high flexibility in a functionally relevant protein area. SLICE enabled detection of two distinct binding areas (Figure 4D) that have both been reported to accommodate allosteric ligands in X-ray crystallography, validating them as cryptic pockets (PDB ID 8TSB and 8W9A). Taken together, cryptic pocket formation occurred in all investigated systems by perturbing the local intramolecular contacts of functional protein segments, upon which the remaining structural rearrangements occurred without steering or the presence of a ligand.

### Successful Simulations Indicate a Hierarchical Mechanism Where Disrupting Specific Close Contacts Unlocks Broader Dynamics Involved in Cryptic Pocket Formation

The structural changes observed in PRMT5 displayed a sequential character in all successful trajectories, where specific contacts required disruption before enabling further dynamics (Figure 5A). First, the salt-bridge-forming D442 and R604 dissociate, enabling F440 to approach V503 and flip to further adopt a crystallographic-like allosteric state. None of our standard MD, REST2-MD, MSMD, SWISH, and metadynamics simulations exhibited this first step of D442-R604 dispersion or subsequent cryptic pocket opening. The trajectories show that benzene molecules do reach the D442-R604 contact but cannot induce its disruption (Supporting Figure S4A). In higher SWISH replicas a different entrance to the pocket appears around N443, I582, and Y633, enabling probes to get closer yet still unable to induce further structural changes (Supporting Figure S4B). The related PRMT6 also forms a salt bridge between its EE loop and local environment, but unlike PRMT5 this does not gate access to the cryptic pocket. Here, H163 first disperses from M373, upon which further rearrangement of H163 shifts the EE loop away and L161 vacates the pocket (Figure 5B).

**Figure 5:**
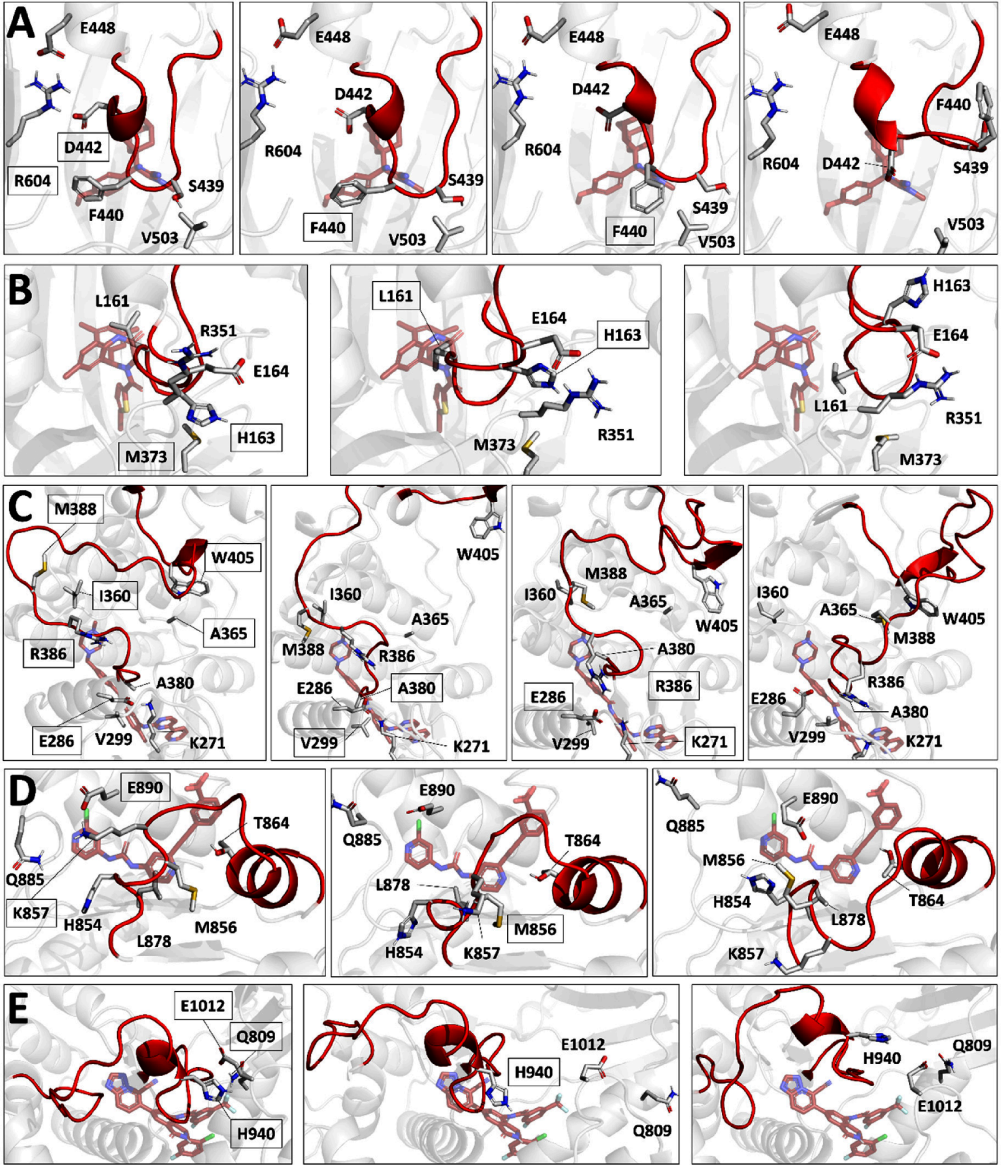
Mechanistic insights into cryptic pocket formation in the targets studied, showing illustrative snapshots from SLICE simulations in which cryptic pockets formed, highlighting the bias region (red), cryptic ligand (transparent red) and key residues (boxed). (A) PRMT5: D442-R604 dissociate first to facilitate F440 movement. (B) PRMT6: M373-H163 disperse followed by L161 displacement. (C) Abl1: rearrangements between M388-I360 and W405-A365 enable R386 to approach E286, facilitated by A380 and V299 displacement, causing R386 to disrupt E286- K271. (D) SMARCA2: E890-K857 disperse and M856 readjusts. (E) PI3Kα: H940 dissociates from E1012 and Q809, enabling the loop/helix segment to vacate the cryptic pocket area.

Abl1 also displayed a sequential character with a salt bridge between K271 and E286 initially preventing the activation loop flip (Figure 5C). Indeed, this flip only occurred when K271 and E286 dissociated. In successful simulation, initial rearrangements between M388-I360 and A365-W405 positioned R386 to approach E286, upon which V299 and A380 dissociated, enabling R386 to approach E286 and disperse E286 from K271. For SMARCA2, the contacts between E890 and K857, as well as E890 and M856 dislocate rapidly to enable the proximal α helix to displace and open the cryptic pocket extension (Figure 5D). Finally, in PI3Kα, one cryptic pocket forms by dissociating E1012 with H940 and Q809, enabling further upward movement of H940 and the helical segment (Figure 5E). The other cryptic pocket displays a less sequential character, as only the displacement between P953 and its contact partners T908 and G194 is sufficient for accessibility. While the cryptic pocket is thus successfully formed and identified in all studied systems (Figure 4), a retrospective look at the crystallized ligands binding these pockets shows that the side chains surrounding these ligands does not necessarily resemble a crystallized arrangement.

## Discussion

Here we investigated the structural basis for cryptic pocket formation in potentially therapeutically relevant proteins driven by extensive conformational changes. Successful simulations indicate an interplay of mechanistic effects for most studied systems, where specific interactions need to be perturbed first upon which further conformational changes to form the cryptic pocket occur undirected.

The need to disperse specific local contacts follows from the observations of a hierarchical cryptic pocket formation in PRMT5, Abl1, SMARCA2, and PI3Kα. In these targets, the broader structural changes involved in cryptic pocket formation only occurred after certain contacts dissociated. In PRMT5 the D442-R604 salt bridge gated conformational flexibility of the EE loop in all simulations. The observations that dispersion of this contact, and subsequent cryptic pocket formation, did not occur in microsecond long unbiased simulations or when treating the EE loop at an effective temperature of 600 K indicate that these dynamics are rare at best in the unliganded conformational ensemble of PRMT5. This follows the observed stability of the EE loop in experimental structure determination. The lack of EE loop dynamics in MSMD and SWISH simulations using 1 M benzene probes furthermore illustrates that small hydrophobic probes and a focus on apolar interactions alone can be insufficient to perturb anchoring contact networks in a broader target scope. The metadynamics, OPES, and SLICE simulations further illustrate the resilience of these interactions, as a considerable local bias was required to achieve loop displacement. Together, these observations indicate that cryptic pocket formation in PRMT5 requires a significant initial enthalpic cost beyond what could be achieved in our setup of established methods. This is in line with previously published studies on different systems where MSMD and SWISH were found to be inadequate in sampling cryptic pocket formation when high energy barriers are involved.^13,27^ Moreover, in this study we observed structural distortion prior to enhanced EE loop dynamics for SWISH and metadynamics (Supporting Figure S2).

Deploying SLICE to enact a strong local perturbation rapidly led to undirected further conformational changes forming the cryptic pocket in all targets tested. This also involved dynamics of interactions that were not part of the original close contacts, indicating that these changes occur as part of the broader conformational ensemble unlocked upon the initial contact disturbance. For instance, in Abl1, the initial rearrangements positioned R386 closer to E286. Without biasing any contacts involving R386 or K271, or the presence of a ligand, this subsequently led R386 to dissociate K271 and E286 upon which the activation loop flipped. It agrees with previous mechanistic studies on the activation loop flip in Abl kinase that the formation of a salt bridge between E286 and R386 is involved.^28^ The observed involvement of salt bridges here, as well as for PRMT5, also indicates that salt bridge dynamics can play a role in governing extensive structural changes. This is congruent with recent work on intrinsically disordered proteins, where structural flexibility and shifting between distant conformations could be mapped to salt bridge behavior.^29^ Collectively, our observations illustrate that resilient contact networks to structurally lock functional protein segments in place occur across several drug targets, and that disrupting these interactions unlocks greater flexibility featuring extensive dynamics to form cryptic pockets.

We opted to define contacts for bias in SLICE based on distance (side-chain COMs within 4.5Å), as this enabled the incorporation of lipophilic interactions that are governed by proximity. To prevent redundant inclusion of multiple carbon–carbon or carbon–hydrogen contacts per residue pair, the side-chain COM was chosen as a central point per residue ensuring that each residue–residue pair would only be considered for bias once. While this definition has been successful for the systems described here, it poses two further considerations. First, it requires a distance cutoff to determine which interactions to include. Although there is no direct limit to the number of interactions to include, a greater number translates to a greater total bias in the system and therefore an increased risk of protein denaturing effects. Conversely, a low number of contacts may not sufficiently perturb anchoring contacts and thereby limit the accessible conformational dynamics. Consistently across the various systems studied here, we found that a cutoff of 4.5Å provides a good balance between these opposing demands, whereas cut-offs of 4.0Å or 5.0Å select either none or redundantly many contacts (Supporting Table S1). For PRMT5, we indeed found that running SLICE with a distance cutoff of 4.0Å did not lead to cryptic pocket formation. A cutoff of 5.0Å did open the cryptic pocket, but it also opened the protein to a greater degree in general, leading to partial structural distortions in the trajectories and consequently a coarser pocket definition (Supporting Figure S5). The effect on computational cost was more limited, with the 4Å simulations (1 contact) taking on average ∼ 50 h wall-time compared to ∼ 55 h for the 5Å (14 contacts) simulations (times per replica, 3 replicas per approach).

A second consideration in our contact definition is that it assigns equal value to all interaction types. As previously discussed, however, our results indicate that for instance salt-bridges may play a more critical role in cryptic pocket formation. To further investigate this, we reperformed the PRMT5 simulations by defining contacts based on 3 criteria: (i) including all H-bond and salt-bridge interactions found in the starting structure with Schrödinger Maestro (v14.3.129),^30^ (ii) including only the H-bond and salt-bridge interactions between side-chains (no backbone) from this list, and (iii) including only salt-bridges from this list. We found that the latter did not sample cryptic pocket formation in PRMT5, while the first two did (Supporting Figure S6). Accordingly, for PRMT5, the cryptic pocket can be found both by distance-based and more chemically rooted interaction definitions, illustrating that protein–protein contacts can be instructive CVs to sample cryptic pocket formation.

Selecting protein conformations from the pool of newly sampled states for further efforts commonly involves plotting the free energy along a CV to identify low-energy conformers. Nevertheless, we show that the goal of identifying ligandable cryptic pockets, even when extensive dynamics are involved, can be achieved by applying SiteMap^26^ on the SLICE conformations. One attribute for a binding pocket to be considered druggable is its ability to elicit a functional effect on the target protein. When faced with a pool of candidate pockets from, e.g., large library screening, it is therefore recommendable to focus further efforts on a pocket that is spatially or energetically related to an endogenous ligand binding site, a cofactor site, or enzyme active site.^5^ In this regard, the current approach does the same by focusing pocket identification on a region that impacts protein function, i.e., proximal to an enzyme active site (PRMT5, PRMT6, Abl1) or validated druggable pocket (SMARCA2). The only practical difference is that the region of interest is defined at the start here, rather than at the end. While there is thus no reliance on prior information regarding ligands, cryptic pocket location, or protein conformation of interest, and SLICE can be used to explore conformational flexibility of any protein region, it will be most instructive to focus on regions of functional or mechanistic relevance. This distinguishes SLICE as a method to explore cryptic pockets proximal to a region of interest, compared to methods like MSMD and SWISH, which first search for pockets across the entire protein and subsequently narrow down to a region of interest for further investigation. Accordingly, SLICE is not intended to replace any existing tools to study cryptic pockets, but rather to broaden the scope of structural changes that can be considered early in drug discovery campaigns. If biological knowledge on the protein under investigation highlights a particular region as functionally relevant, SLICE can efficiently explore the conformational dynamics of that region to investigate if any new pockets form that can be leveraged to lock this region in a different state. Conversely, if no such region is highlighted, a global searching approach like MSMD or SWISH can be more suitable. Where MSMD can highlight cryptic pockets in solvent-accessible areas, and SWISH can induce protein opening motions to enable cosolvent access to buried regions, SLICE can unlock large-scale structural plasticity even in areas that are poorly accessible and gated by resilient local interactions. Thus, SLICE can also function as a secondary approach to further explore a candidate pocket region that emerges in MSMD or SWISH but is insufficiently accessible there to accommodate drug-like molecules. While the systems studied here mostly involved contiguous loops without secondary structure, there is no principal limitation to the regions to which SLICE can be applied. Still, in case of secondary structure disruption one can consider lowering the initial barrier height or using a contact map with an upper wall to limit structural variations within the secondary structure element.^31^ Supporting Figure S7 shows a decision tree to guide method selection for cryptic pocket searching.

Although we have shown SLICE to be successful for systems and regions known to display transitions, an important further question is whether SLICE can also distinguish between false positives and true cryptic pockets. A system that is often considered as a “negative control” in this regard is ubiquitin.^12,32^ This protein has been studied extensively in NMR, which has not revealed any cryptic pockets. Running SLICE on 3 different loops across the protein (PDB ID 2LJ5, 3 replicas, 100 ns each, for all bias regions) did not produce any cryptic pockets with high SiteScore and DScore (both >1.0) values for ubiquitin, illustrating an ability to discern between true cryptic pockets and false positives using druggability scores (Supporting Figure S8). It can also be interesting to address this question from an energy perspective. However, the approach presented here primarily focuses on exploring different protein configurations rather than converging the free energy of pocket opening and because of the “exploration-convergence trade-off” this makes it difficult to obtain a reliable estimate of the energy penalty associated with cryptic pocket formation via this approach.^23^ Nevertheless, using a slightly adapted biasing protocol (SLICE-MetaD), and incrementally increasing the deposited bias per contact in SLICE, the energetic cost can be indirectly probed (Supporting Figure S9). This showed that only the previously validated cryptic pocket in PRMT5 opened within the tested energy range, while no substantial structural rearrangements were detected for the negative control, ubiquitin.

Finally, although cryptic pocket formation was observed in all studied proteins, the side chains lining these pockets did not necessarily adopt a crystallographic arrangement. This indicates that a final rearrangement is still required to complement the specific allosteric ligand once the cryptic pocket is accessible. Indeed, different allosteric ligands binding the same cryptic pocket in PRMT5 (PDB ID 6UXX vs 6UXY) or in PI3Kα (PDB ID 8TGD vs 8TSB) exhibit different surrounding side chain orientations despite the pocket being accessible in all these structures. Since SLICE does not include ligands, a decision made purposely to prevent the need for prior knowledge, it does not allow for this local refinement. This illustrates that identifying a protein conformation with an accessible cryptic pocket and obtaining full ligand-protein complementarity are separate considerations. Accordingly, ligand exploration and experimental validation is still required in the drug discovery context once the cryptic pocket is detected. To further illustrate this, an overview of the backbone and all-atom RMSD of the cryptic pocket residues in PRMT5 and PI3Kα is provided in Supporting Figures S10 and S11 and additional 100 ns standard MD simulations with the four different ligands from PDB IDs 6UXX, 6UXY, 8TGD, and 8TSB were performed. This indeed showed that the all-atom RMSD of cryptic pocket residues is induced down in the presence of a ligand (Supporting Figures S10 and S11). Furthermore, the stability of the ligands in the pocket, illustrated by their overlap with the cocrystallized complex at the end of the simulation (Supporting Figures S10 and S11), shows that SLICE states can accommodate drug-like ligands. While ligands would not be known in a prospective design setting, the effect of ligand presence on stabilizing lower RMSD conformations indicates that there could also be merit to inserting particles acting as a placeholder for ligands during SLICE exploration.^33^

## Conclusions

The current work illustrates that functional protein segments can be structurally locked by protein contact networks and that disrupting these interactions can lead to extensive structural rearrangements upon which ligandable cryptic pockets emerge. The currently presented method SLICE can exploit these conceptual insights to provide a better understanding of the structural plasticity around functional protein segments and aid in identifying cryptic pockets.

## Methods

### System Preparation

All molecular dynamics simulations in this work were performed using GROMACS^34^ version 2021.4 patched with PLUMED^35^ version 2.8.0 along with the DES-Amber1.0 force field^36^ and TIP4P-D waters.^37^ To limit bias arising from different research groups and crystallization conditions, the highest resolution PRMT5 structure resolved by Merck & Co., who solved the allosteric states, showing the EE loop in its default conformation was selected as starting structure: PDB ID 7KIC. While PRMT5 exists as a hetero-octamer (PRMT5)_4_(MEP50)_4_ only the monomeric 7KIC unit was considered and further trimmed to include only residues 294–637 to cut required simulation times. This structure was subsequently capped with an ACE residue at the N-term using PyMOL (v3.0.0), stripped of the cocrystallized ligand to obtain an *apo* state, and prepared for simulation using Schrödinger Maestro (v12.9) Protein Preparation Wizard at default settings. The resulting protonated structure was then solvated (0.15 M ion concentration), energy minimized in GROMACS^34^ over 50 000 steps (steepest descent algorithm, 100 kJ/mol/nm tolerance), and equilibrated in three steps. This comprises an initial 1.0 ns NVT simulation (V-rescale thermostat,^38^ τ=0.1 ps) and subsequent 5.0 ns NPT simulation (V-rescale thermostat,^38^ τ=0.1 ps, Berendsen barostat,^39^ P = 1 atm, τ=0.5 ps) using heavy-atom harmonic position restraints (k = 1000 kJ/mol/nm), followed by a final 5.0 ns NPT simulation (V-rescale thermostat,^38^ τ=0.1 ps, C-rescale barostat,^40^ P = 1 atm, τ=1.0 ps). In all cases, a reference temperature of 300 K was set, defining the protein and (co)­solvent including ions as two separate temperature coupling groups, long-range electrostatics were considered using the particle mesh Ewald^41^ method (r = 1.2 nm), and a 2 fs time step was used with the LINCS algorithm^42^ to constrain H-bond stretching. Unless specifically stated, the final structure produced by this NPT simulation was used as starting structure for all production simulations across all protocols.

### Unbiased MD

Unbiased MD production simulations were run in the NPT ensemble, using the same parameters described for the second NPT step in the previous section. Three independent replicas of 1 μs each were simulated to account for slow conformational changes.

### REST2-MD

The topology file created during the steps described under system preparation was first processed to obtain a file without “include statements”. Underscores were then added to atom types of all residues to include in the scaling. Here, residues 435–452 were selected to include the alpha helical structure attached to the EE loop. These atom types were subsequently scaled with a factor lambda to resemble behavior for a target temperature of 600 K for only the selected protein region. While REST2 is typically performed in a replica exchange protocol, here only the most scaled replica (target T = 600 K) was considered to evaluate EE loop dynamics with the intent to run the replica-exchange protocol if unfolding occurs. The scaled topology file was then used to run a new equilibration phase, as described before, followed by three independent 1 μs production runs.

### Mixed-Solvent MD

To prepare MSMD simulations, the initial capped and protonated PRMT5 structure was first solvated (0.15 M ion concentration) and energy minimized in GROMACS^34^ (50 000 steps, steepest descent algorithm, 100 kJ/mol/nm tolerance). Next, solvent molecules were replaced with benzene molecules to solvate the protein in 1 M benzene. This cosolvent type and concentration was selected based on earlier reported success to find cryptic pockets with this setup.^12^ Parameters for the benzene molecules were obtained using Gaussian 16 and the Amber GAFF-2 force field^43^ with RESP charges. A repulsive potential was also added to prevent phase separation between the benzene and water molecules. The system was then subjected to the same equilibration process described above, using the protein and benzene as one temperature coupling group, followed by three independent 500 ns MD simulations.

### SWISH

SWISH simulations were run in a standardized replica-exchange protocol using eight parallel replicas. A global topology file was first created as described for MSMD to obtain a solvated PRMT5 system with 1 M benzene probes. Eight individual topology files were then generated from this, each with a unique scaling factor value (λ) uniformly distributed ranging from 1.00 to 1.35. Each replica was then individually subjected to the equilibration protocol described above followed by 500 ns production runs allowing replica exchanges. To minimize the likelihood for protein unfolding, a contact map restraint was included in the production run. The upper wall limit for this restraint was determined by analyzing fluctuations in the contact map value in unbiased simulations.

### Metadynamics

Using the same topology file and starting structure as for the unbiased production runs, a PLUMED-readable file was created to provide instructions for metadynamics. First, center of mass (COM) calculation for the EE loop was instructed by defining COM ATOMS = 2262–2422. The components of the Cartesian coordinates for this COM were then decomposed and set as three individual CVs with sigma = 0.01, height = 0.5, and pace = 500. Three independent 200 ns production runs were then simulated using these instructions for PLUMED^35^ and the same simulation parameters described for the unbiased protocol.

### OPES and SLICE

The initial OPES protocol used the same steps described for metadynamics to define the Cartesian coordinate components of the EE loop COM. These components were defined as arguments for the OPES_METAD^22^ keyword with barrier set to 250 kJ/mol and pace at 500 steps to instruct PLUMED^35^ to apply an OPES^22^ bias to these CVs. Three independent production runs of 100 ns were subsequently simulated using these instructions for PLUMED^35^ and the parameters described for the unbiased protocol. For the final SLICE protocol, the initial capped and protonated PRMT5 structure was freshly solvated (0.15 M ion concentration) and energy minimized in GROMACS^34^ (50 000 steps, steepest descent algorithm, 100 kJ/mol/nm tolerance). Using the Cartesian coordinates stored in the resulting PDB structure, along with the atom masses stored in the accompanying topology file, the COM of the side chains for all residues were then calculated in Bash, along with the distance between each pair of COMs. Atom names N, CA, C, HA, H, and O were not considered in this calculation to ensure only side chain atoms were included. This distance list is then automatically filtered to only retain pairs where the side chain COM of a residue outside the region of interest lies within 4.50Å of the side chain COM of a residue inside the region of interest. Each close contact is then directly stored as an individual COORDINATION CV in a PLUMED-readable file, and subjected to an OPES Explore^23^ bias using the OPES_METAD_EXPLORE keyword (barrier = 50 kJ/mol, pace = 5000 steps) in three independent 100 ns production runs. The barrier height and pace were lowered with respect to the initial OPES protocol to account for the more aggressive exploration of OPES Explore.^23^ For PRMT5, the close contacts found were L437-Y468, S439–V503, F440–S470, and D442-R604.

### Pocket Detection with SiteMap

To prepare for cryptic pocket detection, the first 100 ns of three independent unbiased MD simulations were stored as 200 separate PDB files (1 frame per 0.5 ns). The same was done for the three 100 ns independent SLICE simulations using the close contact CVs described above. Each frame was then prepared using Schrödinger’s Protein Preparation Wizard and subjected to a SiteMap^26^ calculation through the command line using reportsize 150 and nthresh 10. The detected pockets were then stored separately per frame. All “site points” found in any frame of the unbiased trajectories were then combined and stored as a single PDB file, using PyMOL (v.3.1.4.1). Next, the SiteMap results for all frames of the first biased replica were loaded into PyMOL, as well as the PDB file containing all unbiased site points. To retain only cryptic pockets, any sites where more than 20% of the site points lie within 2.0Å of a site point from the unbiased trajectories were then removed. To focus on druggable pockets, only detected pockets with SiteScore >1.0 and DScore^26^ >1.0 were subsequently kept. The remaining pockets were then stored in a PDB file, and the process was repeated for the second and third biased replica. Final results were then visualized in PyMOL by opening the original protein structure along with the three PDB files containing the site points passing all aforementioned filters.

### Additional Systems

For additional systems the same protocols for preparation, biasing, close contact CV definition, and pocket detection were applied as described above. For PRMT6, the AF2 model AF-Q96LA8-F1-model_v4 was used as starting point, trimmed to include residues 53–375 and N-terminally capped. Residue 155–165 (PRMT6’s EE loop) was set as region of interest, and the following automatically selected close contacts were biased: E155-R66, G160-A321, L161-F292, H163-M373. For Abl1, PDB ID 2V7A was used as starting point, keeping only chain A, capping both termini, setting residue 379–408 (the activation loop) as region of interest, and using the following automatically selected close contacts as CVs: A380-V299, A380-L370, G383-E286, M388-I360, T394-K415, A395-N414, W405-A365, A407-S420, A407-W423. For SMARCA2, AF2 model AF–P51531-F1-model_v4 was used as starting point, resembling PDB ID 6EG2, trimming to include only residues 705–955, capping both termini. Residue 852–860 (lining the known binding pocket) was set as region of interest, and the following automatically selected close contacts were biased: G853-L878, H854-Q885, M856-T864, K857-E890. Finally, for PI3Kα the AF2 model AF–P42336-F1-model_v4 was used as starting point, resembling PDB ID 8TS7, trimming to include only residues 765–1051, capping both termini, setting residue 931–957 (loop with missing electron density in PDB ID 8TS7) as region of interest, and using the following automatically selected close contacts as CVs: F934-T813, G935-Q809, G935-D810, H940-E1012, V952-M1043, V952-A1046, P953-T908, P953-G914, and L956-H1047.

## Supplementary Material



## Notes

RMSD plots of PRMT5 for the simulation methods tested, illustrative snapshots of the structural distortions observed in some PRMT5 simulations, as well as of the DFG-flip observed in Abl1, benzene occupancy around the cryptic pocket in PRMT5, pocket visualizations obtained in SLICE using different contact definitions, SLICE results on ubiquitin as negative control, an investigation of the energetic cost of cryptic pocket opening, and a decision tree to guide method selection for cryptic pocket searching (PDF)

## Abbreviations

AF2: AlphaFold 2
CV: collective variable
COM: center of mass
MD: molecular dynamics
MSMD: mixed-solvent MD
OPES: on-the-fly probability enhanced sampling
PDB: protein databank
PRMT: protein arginine methyltransferase
REST: replica-exchange with solute tempering
RMSD: root mean squared deviation
SLICE: sampling by local interaction-guided conformational exploration
SWISH: sampling water interfaces through scaled Hamiltonians
