# Supplementary material for: Exploring the Structural Basis of Cryptic Pocket Formation Driven by Extensive Protein Conformational Changes in Drug Targets
Source: J Chem Theory Comput. 2026 Mar 4;22(6):2714–24. doi: 10.1021/acs.jctc.5c02016 (PMC13019620; doi:10.1021/acs.jctc.5c02016)
Supplement: Supplementary file 1 [file ct5c02016_si_001.pdf]

Supporting information

Exploring the Structural Basis of Cryptic Pocket Formation Driven by Extensive Protein Conformational Changes in Drug Targets

Martijn P. Bemelmans[1,2]*, Alberto Borsatto*[2,3]*, Simone Marsili*[4]*, Francesco L. Gervasio*[2,3,5]**, and*

Vineet Pande[1]*

[1]Computer-Aided Drug Design, In Silico Discovery, Therapeutics Discovery, Johnson & Johnson Innovative Medicine, Turnhoutseweg 30, 2340 Beerse, Belgium

[2]School of Pharmaceutical Sciences, University of Geneva, Rue Michel Servet 1, Geneva, 1206, Switzerland

[3]Swiss Bioinformatics Institute, University of Geneva, Geneva, 1206, Switzerland

[4]Computer-Aided Drug Design, In Silico Discovery, Therapeutics Discovery, Johnson & Johnson Innovative Medicine, C. Río Jarama, 75, 45007 Toledo, Spain

[5]Chemistry Department, University College London (UCL), WC1E 6BT, London, UK

*Corresponding authors. Email: francesco.gervasio@unige.ch; vpande@its.jnj.com

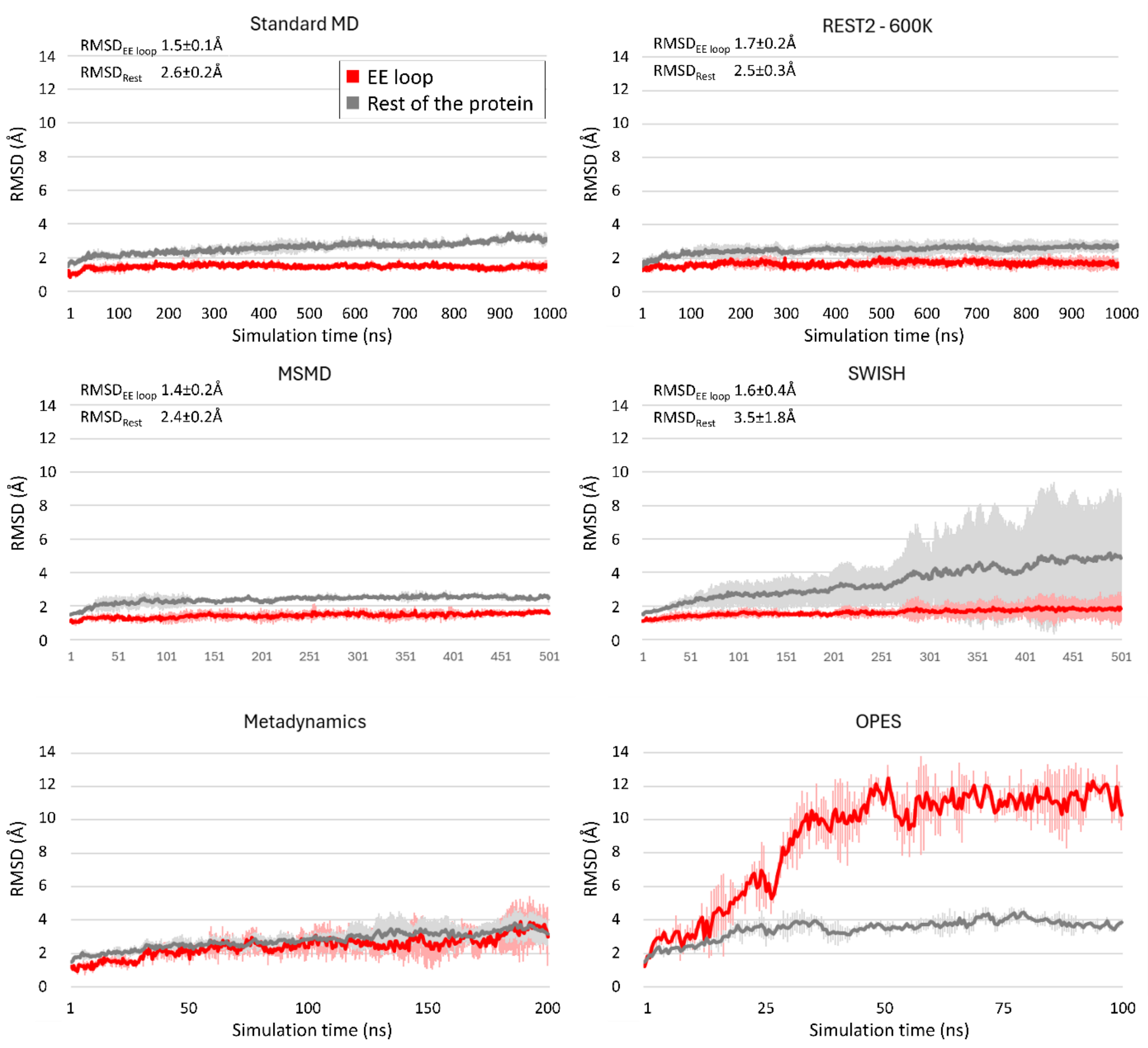

Figure S1: Full protein and EE loop dynamics of PRMT5 across simulation methods. The average root mean squared deviation (RMSD) of the EE loop (residue 435-445, thick red lines) or the rest of the protein (thick grey lines) is shown per simulation method as the simulation time progresses. All simulations were performed in triplicate, with the grey and red areas around the average lines showing the standard deviation across replicas.

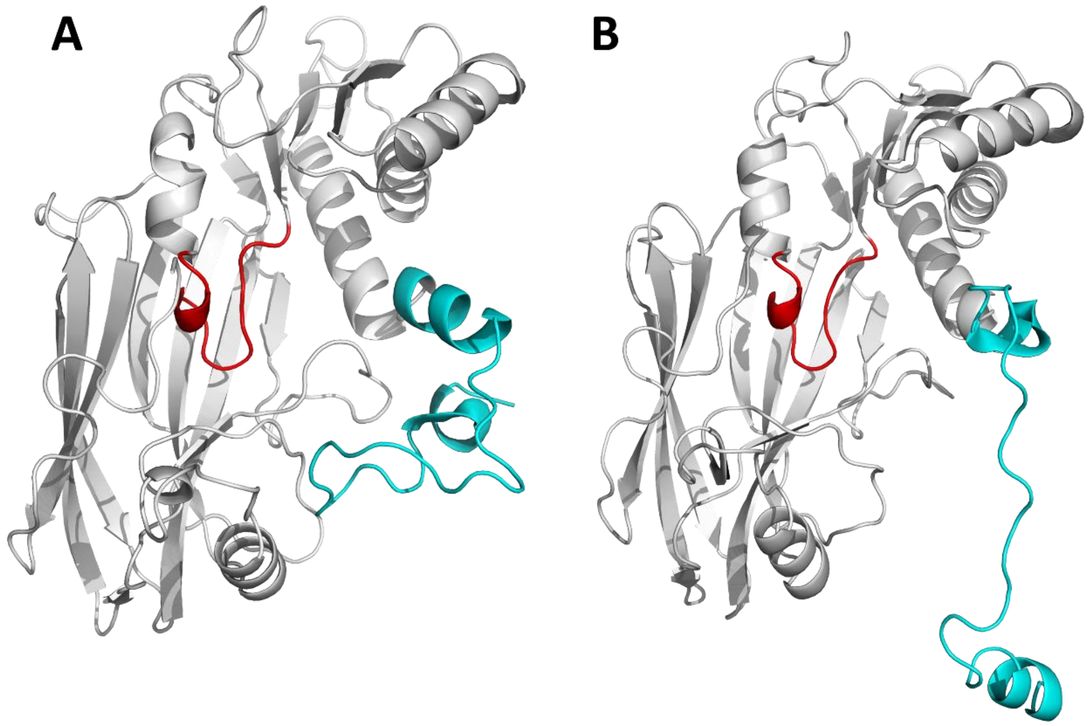

Figure S2: Structural distortions observed in simulations with established methods. **A**) Shows an example state illustrating structural distortion in a metadynamics simulation occurring while EE loop movement remained comparable to the rest of the protein, and **B**) depicts such a state in a SWISH simulation. While further optimization of the metadynamics and SWISH approaches may lead to satisfactory results, the observation that partial structural distortion occurs prior to enhanced EE loop movement dampens this hypothesis.

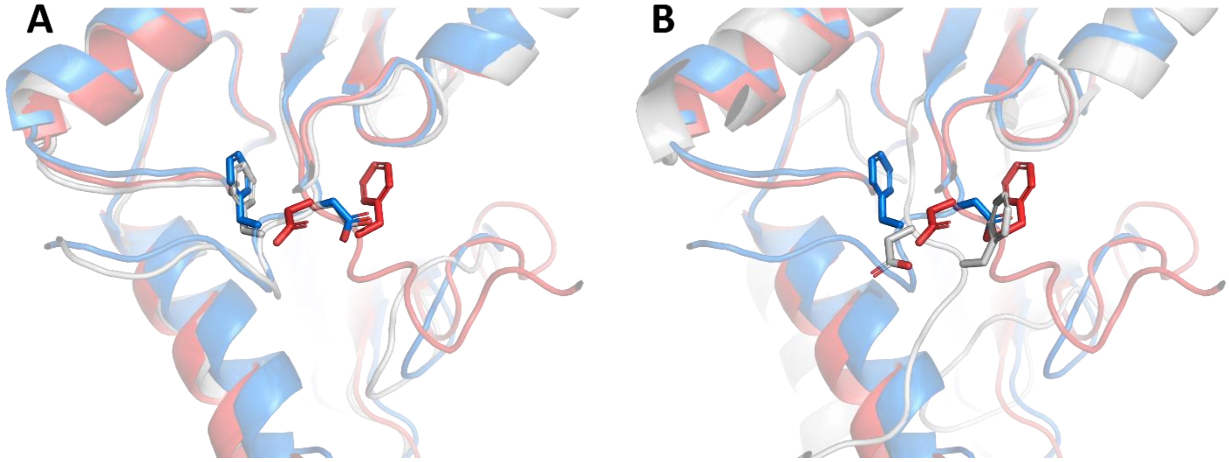

Figure S3: The DFG flip in Abl1 kinase. The aspartate, phenylalanine, and glycine residues in their corresponding structure are shown adopting a "DFG-in" (blue, PDB ID 2V7A), "DFG-out" (red, PDB ID 1IEP) and sampled SLICE (white) state. **A**) shows the starting SLICE state (white), resembling the DFG-in state (blue), while **B**) depicts a state from the last 10ns of a SLICE simulation (white), resembling a DFG-out conformation (red). While this extent of DFG flip did not occur in the first three replicas of SLICE, it shows that additional replicas (in this case 7) can sample a full and spontaneous DFG flip.

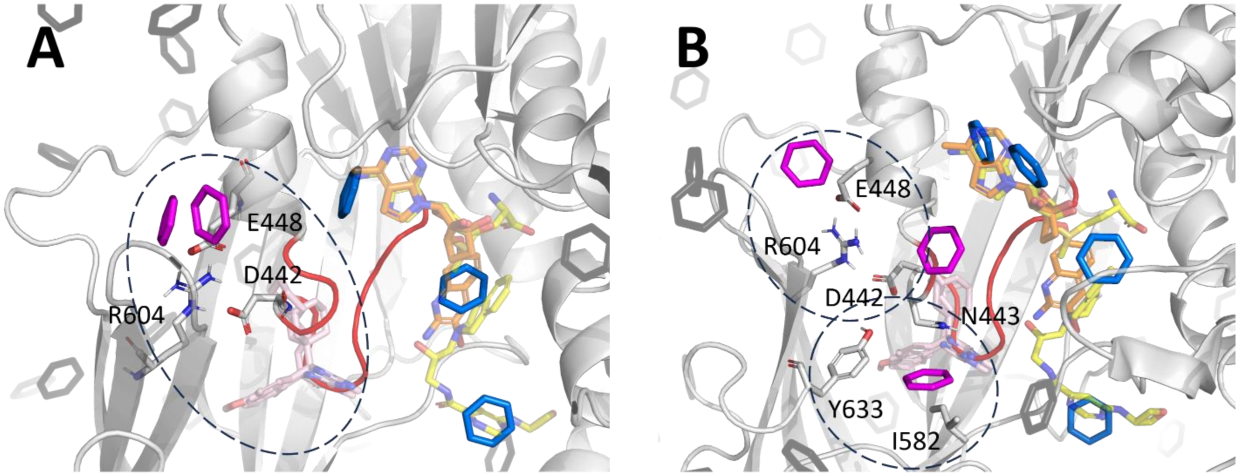

Figure S4: Benzene occupancy around the cryptic pocket in PRMT5. Both panels show benzene probes nearing the cryptic pocket in magenta, those occupying the cofactor and substrate binding site in blue, and others in black. The EE loop (red), cryptic ligands (light pink), example binders of the cofactor (orange) and substrate (yellow) binding pocket, and key residues (white) are highlighted. **A**) shows snapshot 501 (500ns) of the second replica of the MSMD run. **B**) shows snapshot 225 (224ns) of replica 6 of the SWISH simulation. In MSMD (**A**) benzene probes (magenta) approach the cryptic pocket from above but lodge at the salt bridge interactions between R604, D442, and E448, unable to disperse this contact and reach the pocket. The induced protein opening motions in SWISH (**B**) enable benzene probes (magenta) to approach the cryptic pocket through both a top and bottom entrance. Still, the benzene probes are unable to protrude the protein contacts between R604, D442, and E448, or between Y633, I582, and N443 to reach the cryptic pocket. In both simulations, the probes (blue) do cover the readily accessible substrate and cofactor binding site.

Table S1. Number of contacts considered per distance cut-off for each target. The table shows that low distance thresholds (≤4.0Å) select only one or no contacts, leading to limited bias applied to the system, whereas a threshold of 5.0Å can select many contacts leading to greater total bias and risking structural distortion.

Target	Distance threshold (Å)	Number of contacts	Contact IDs	Contact distance (Å)
PRMT5	3.5	0	-	-
	4.0	1	S439-V503	3.7
	4.5 (default)	4	S439-V503<br>F440-S470<br>D442-R604<br>L437-Y468	3.7<br>4.1<br>4.4<br>4.4
	5.0	14	S439-V503<br>F440-S470<br>D442-R604<br>L437-Y468<br>F440-G553<br>E435-K333<br>A441-F555<br>E435-L371<br>A441-S470<br>L445-I635<br>L445-V611<br>D442-S470<br>E444-P314<br>D442-F521	3.7<br>4.1<br>4.4<br>4.4<br>4.6<br>4.6<br>4.7<br>4.7<br>4.8<br>4.8<br>4.8<br>4.9<br>5.0<br>5.0
PRMT6	3.5	0	-	-
	4.0	1	G160-A321	3.8
	4.5 (default)	4	G160-A321<br>H163-M373<br>L161-F292<br>E155-R66	3.8<br>4.2<br>4.4<br>4.4
	5.0	11	G160-A321<br>H163-M373<br>L161-F292<br>E155-R66<br>L161-A189<br>Y159-V238<br>M157-V294<br>E164-Y47<br>S165-R351<br>L161-L191<br>G160-I290	3.8<br>4.2<br>4.4<br>4.4<br>4.6<br>4.7<br>4.7<br>4.7<br>4.8<br>4.8<br>5.0
Abl1	3.5	0	-	-

	4.0	2	G383-E286<br>T394-K415	3.8<br>4.0
	4.5 (default)	9	G383-E286<br>T394-K415<br>A380-V299<br>M388-I360<br>A395-N414<br>A407-W423<br>A407-S420<br>A380-L370<br>W405-A365	3.8<br>4.0<br>4.0<br>4.2<br>4.2<br>4.3<br>4.3<br>4.3<br>4.4
	5.0	24	G383-E286<br>T394-K415<br>A380-V299<br>M388-I360<br>A395-N414<br>A407-W423<br>A407-S420<br>A380-L370<br>W405-A365<br>S385-H361<br>W405-P439<br>K404-P439<br>P408-W423<br>M388-N358<br>P408-W478<br>K404-V448<br>A399-N414<br>S385-V289<br>W405-R367<br>W405-V427<br>L384-R362<br>F382-M290<br>V379-A350<br>D381-N368	3.8<br>4.0<br>4.0<br>4.2<br>4.2<br>4.3<br>4.3<br>4.3<br>4.4<br>4.6<br>4.7<br>4.7<br>4.7<br>4.7<br>4.7<br>4.7<br>4.8<br>4.8<br>4.8<br>4.8<br>4.9<br>4.9<br>4.9<br>5.0
SMARCA2	3.5	0	-	-
	4.0	1	K857-E890	3.999
	4.5 (default)	4	K857-E890<br>M856-T864<br>H854-Q885<br>G853-L878	3.999<br>4.052<br>4.284<br>4.426
	5.0	6	K857-E890<br>M856-T864	3.999<br>4.052

			H854-Q885<br>G853-L878<br>R855-P781<br>M856-F897	4.284<br>4.426<br>4.786<br>4.900
PI3Ka	3.5	0	-	-
	4.0	1	V952-A1046	3.7
	4.5 (default)	9	V952-A1046<br>P953-G914<br>V952-M1043<br>P953-T908<br>H940-E1012<br>L956-H1047<br>G935-Q809<br>F934-T813<br>G935-D810	3.7<br>4.3<br>4.4<br>4.4<br>4.4<br>4.4<br>4.4<br>4.4<br>4.5
	5.0	18	V952-A1046<br>P953-G914<br>V952-M1043<br>P953-T908<br>H940-E1012<br>L956-H1047<br>G935-Q809<br>F934-T813<br>G935-D810<br>H940-D1018<br>E950-K1024<br>H931-Y904<br>L956-L961<br>H931-I921<br>I932-M922<br>K941-D915<br>H931-R916<br>V952-F909	3.7<br>4.3<br>4.4<br>4.4<br>4.4<br>4.4<br>4.4<br>4.4<br>4.5<br>4.5<br>4.6<br>4.7<br>4.7<br>4.8<br>4.8<br>4.8<br>4.9<br>5.0

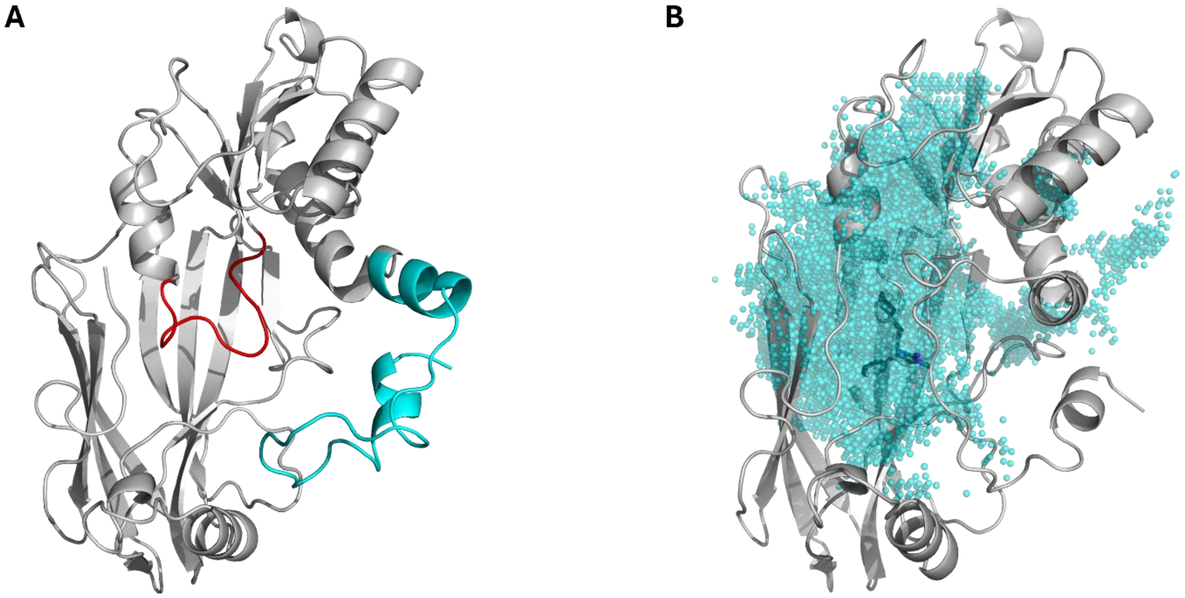

Figure S5. Cryptic pocket definition in PRMT5 using a 5Å distance cut-off in SLICE. A) Shows an example of how the greater total bias associated with a larger number of included contacts (14 at 5Å versus 4 at 4.5Å) can elicit some structural distortions (cyan). The EE loop is highlighted in red. **B**) Depicts the cryptic pocket definition from SLICE at a 5Å contact distance threshold. While this includes the cryptic binding region, the elevated bias leads to further protein opening and thus a less well-defined pocket.

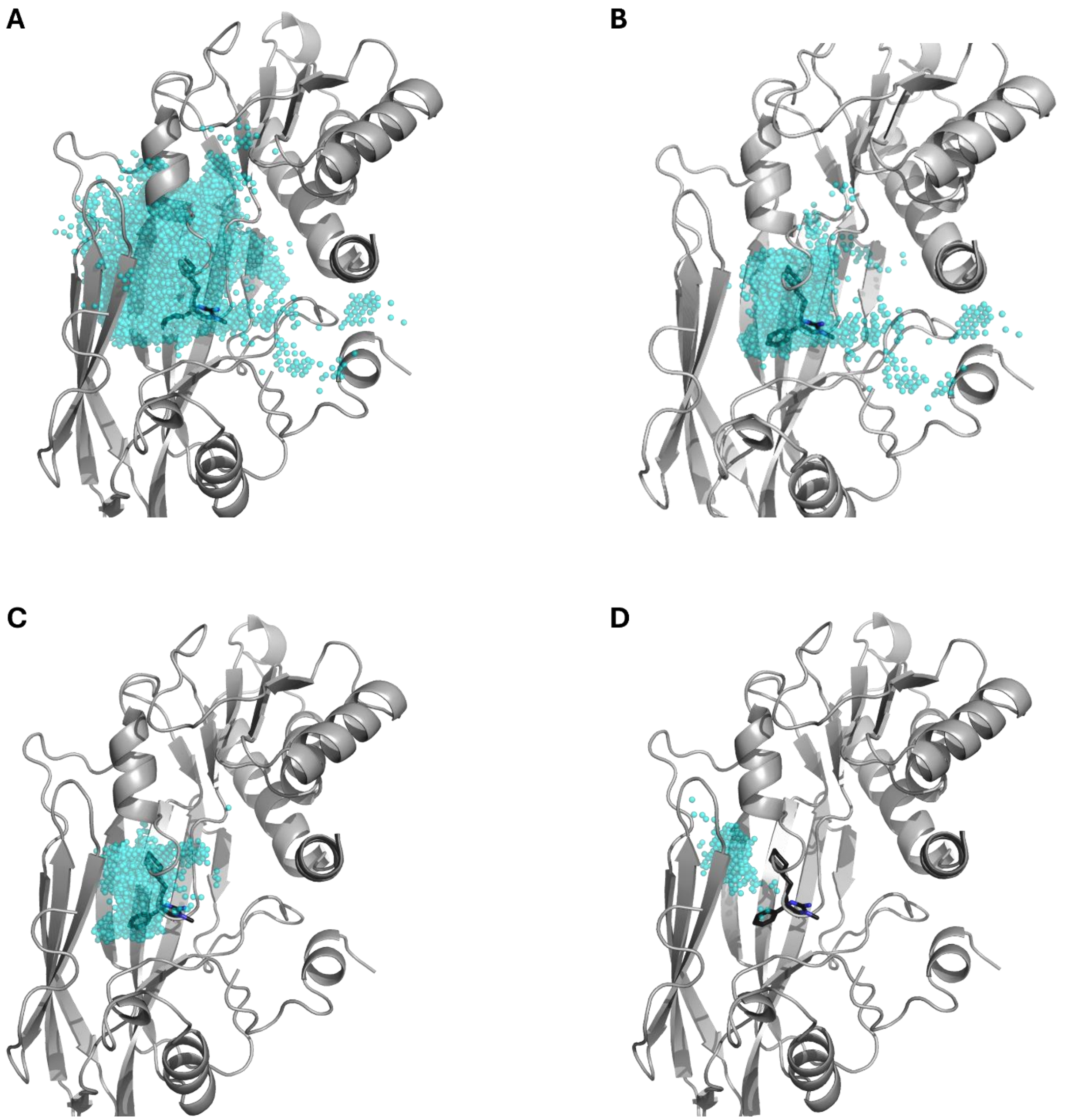

Figure S6. Cryptic pocket definitions in PRMT5 using protein-protein interactions defined with Maestro for bias. A) Shows the pocket definition overlapping regions highlighted in all 3 replicas when using all protein-protein interactions found in Schrödinger Maestro. The interactions included for bias in this contact definition are E435-K333, S439-F580, F440-F471, D442-S470, D442-R604, L437-W579 (6 total interactions). **B**) Shows the pocket definition from the first replica using the same interactions as in A, providing a notable overlap with the cryptic ligand. **C**) Illustrates an overlap of pockets found in all 3 replicas using only the contacts from Maestro that do not involve backbone atoms: E435-K333, D442-S470, and D442-R604. This definition also enables uncovering the cryptic pocket. **D**) Shows an overlap of all pockets found in the 3 replicas using only the salt-bridge contacts found in Maestro: E435-K333 and D442-R604. These interactions were insufficient in the 3 sampled replicas to aid uncovering the cryptic pocket.

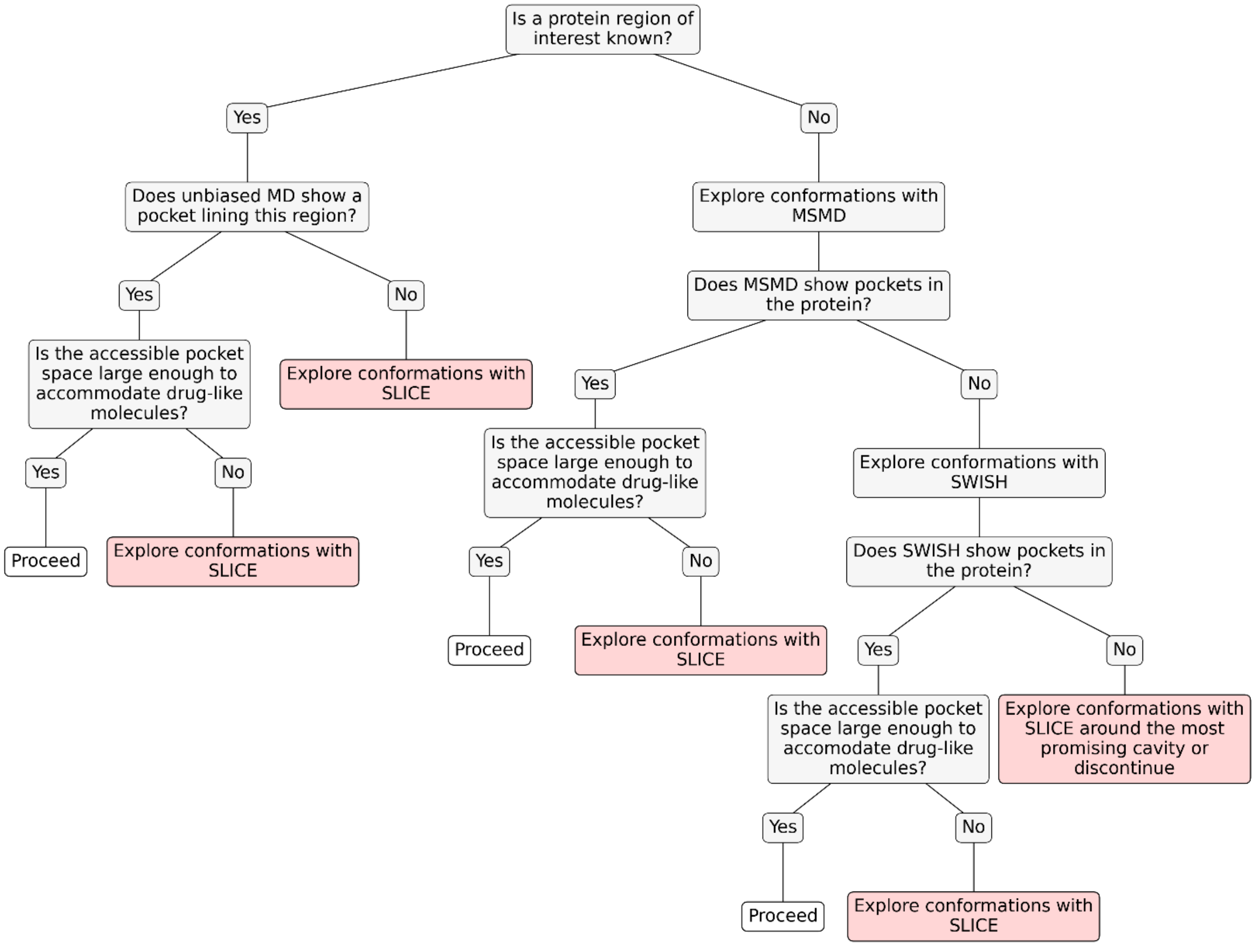

Figure S7. Decision tree to select methods for cryptic pocket searching. To search for cryptic pockets, one can either apply a global searching method like MSMD and SWISH and subsequently narrow down to a region for further exploration or start from a region of interest and apply a local bias method like SLICE to explore pockets proximal to this region. Regions of interest can include structural elements neighboring an enzymatic active site, regions missing density (thus being highly dynamic), or lining known binding pockets. The rationale is that locking such regions in a different state by proximally opening pockets has the potential to affect biological functioning. Accordingly, we envision a good use-case for SLICE to be a scenario where a protein region of interest is known, such as the functional loop in PRMT5. When such a region is not known, MSMD can be more appropriate, though this method may not map pockets that are poorly solvent-accessible, in which case SWISH can be used. Alternatively, if MSMD and/or SWISH help to identify a candidate pocket that is not accessible to drug-like molecules, the neighboring protein region can be used in SLICE for further conformational exploration. When a suitable pocket is found, one can proceed with further computational or experimental validations. Notably, there are numerous other approaches to search for cryptic pockets and this is not an exhaustive list but merely an illustrative overview of possible scenarios to employ SLICE in the context of common other approaches.

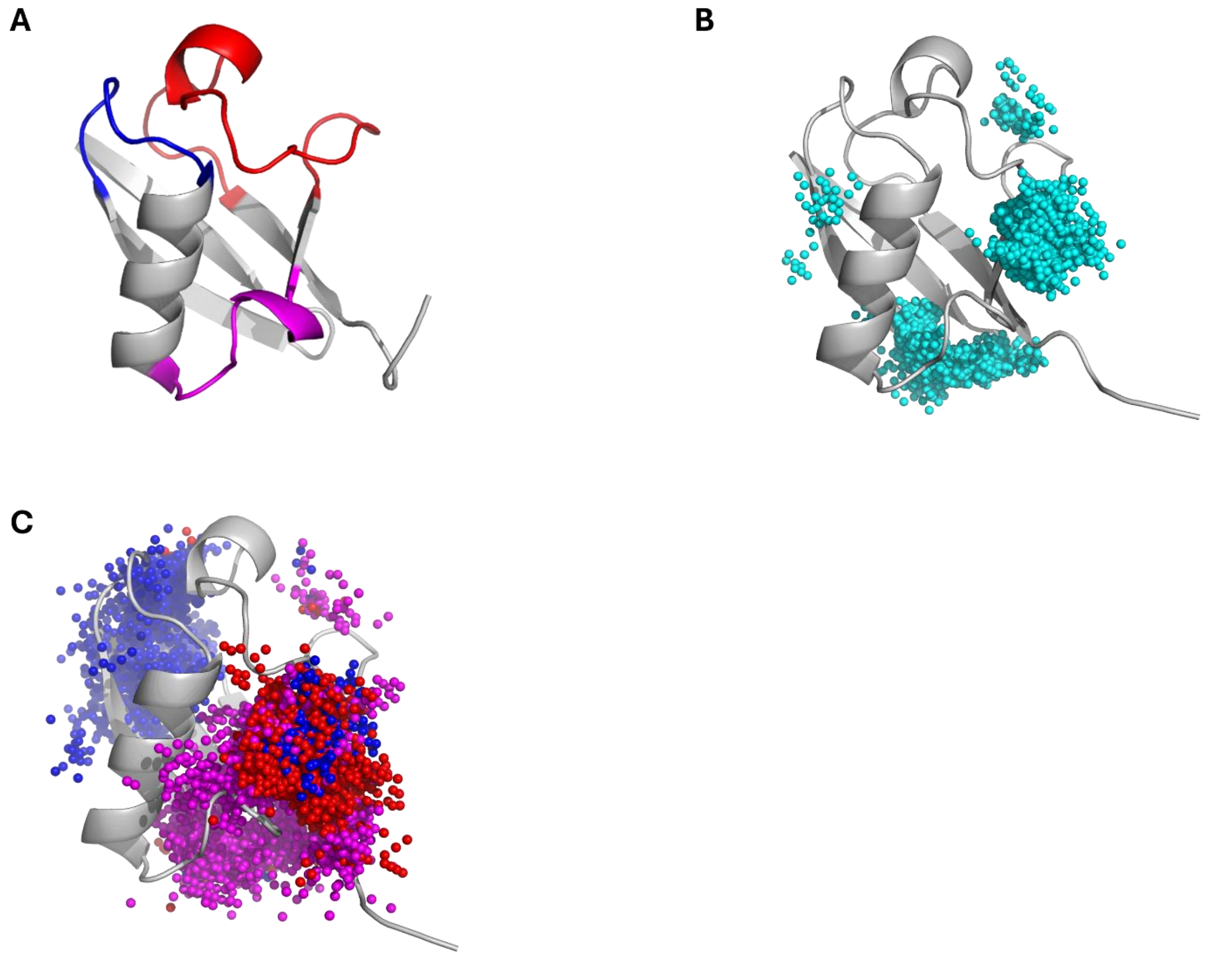

Figure S8. Cryptic pocket exploration with SLICE on ubiquitin. A) Highlights the three regions used to bias with SLICE for cryptic pocket exploration. Residue 17-23 is colored in blue, residue 34-41 in magenta, and residue 44-66 in red. **B**) Depicts the pocket space found by SiteMap in unbiased MD (3 replicas, 100ns each). **C**) Shows an overview of the cryptic pocket space found by SiteMap in the SLICE trajectories. For visualization purposes, the pockets from only one replica per bias region are shown, though each region was simulated in 3 replicas, 100ns each. None of these pockets qualified as ligandable cryptic pockets (SiteScore>1.0 and DScore>1.0, <20% overlap with pockets identified in unbiased MD), thus SLICE does not highlight any pockets for ubiquitin.

SLICE-MetaD

To investigate cryptic pocket opening from an energy perspective, we slightly modified the SLICE protocol to explore how different bias energy scales in OPES influence the opening of the cryptic pocket in PRMT5 and to compare this behavior with a negative control, ubiquitin. We employed the OPES-MetaD biasing scheme to allow finer control over the magnitude of the deposited bias, particularly during the initial stages of the simulation. That is, compared to the OPES Explore biasing scheme used by default in SLICE, which is focused more on exploration rather than convergence. For PRMT5, bias was applied to contacts involving the EE loop region, using a re-equilibrated structure designating F440–S470, D442–R604, L437–Y468, and A441–S470. For ubiquitin, contacts involving loop residues 17–24 (V17–I3 and D21–N25) were selected. The applied bias range spanned from 15 to 50 kJ/mol, and backbone restraints were imposed on non-loop secondary structure elements to limit large scale structural rearrangements that could influence the results. For the same reason, bias deposition was stopped once the selected contacts were disrupted. The resulting structural responses are summarized on the next page.

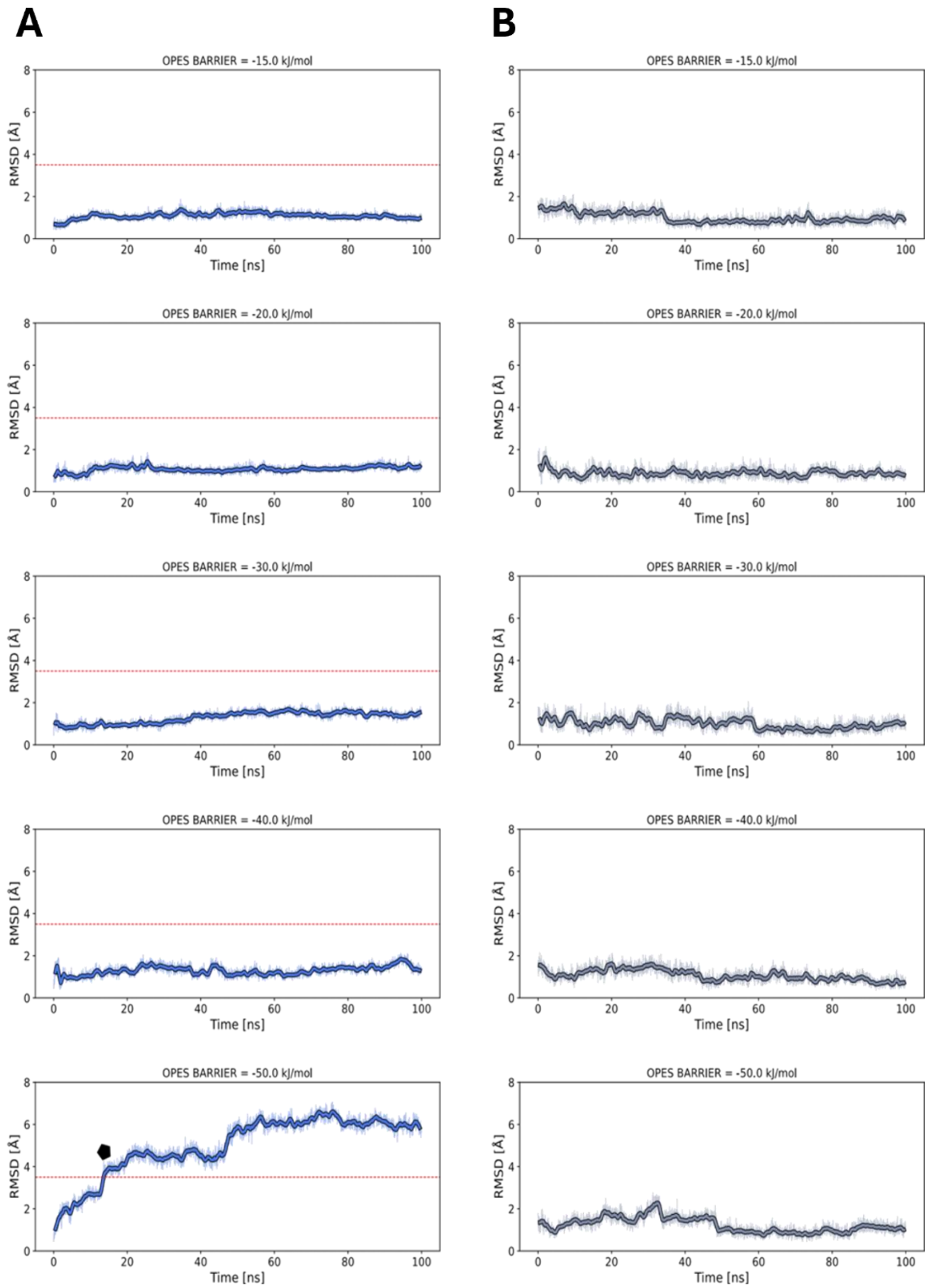

Figure S9. Investigating cryptic pocket opening from an energy perspective. Different bias energy barriers tested using OPES-MetaD to induce the opening of the PRMT5 cryptic pocket (**A**) and of a loop region in ubiquitin (residues 17–24, PDB: 1UBQ) used as a negative control (**B**). Opening events are indicated by black pentagons, solid lines show time-averaged RMSD values (1ns windows), and the shaded areas represent instantaneous RMSD values. The red dashed line represents the reference RMSD value used to consider the PRMT5 cryptic pocket open. The opening process is monitored as a function of simulation time using the RMSD of the loop regions with respect to the corresponding starting structures.

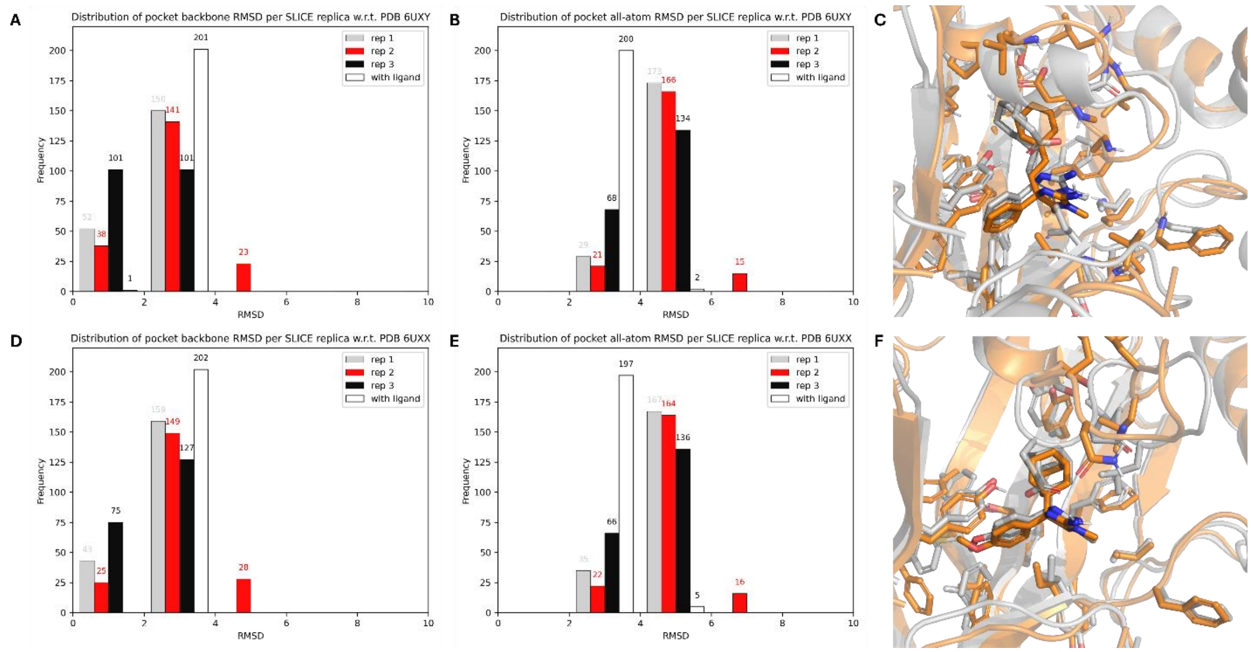

Figure S10. SLICE in the context of the cryptic pocket in PRMT5. A) Shows the distribution of pocket backbone RMSDs for each SLICE replica with respect to PDB ID 6UXY. **B**) Shows the same distribution but considering the all-atom RMSDs. **C**) Depicts the final frame of a 100ns simulation with the cryptic ligand from PDB ID 6UXY (grey) in an open SLICE state (orange). **D**) Shows the distribution of pocket backbone RMSDs per SLICE replica compared to PDB ID 6UXX. **E**) Shows the same distribution but considering the all-atom RMSDs. **F**) Illustrates the final frame of a 100ns simulation with the cryptic ligand from PDB ID 6UXX (grey) in an open SLICE state (orange). The distributions indicate that SLICE visits backbone states that are close to the crystal structure (6UXY or 6UXX) several times across replicas. Presence of a ligand induces the all-atom RMSD down, stabilizing in the 2-4Å range for both 6UXX and 6UXY. Furthermore, both ligands remain stable in their respective SLICE states, illustrated by the overlap with the crystal structures in the last frame of the 100ns simulation. Accordingly, the SLICE states are accommodating to their cryptic ligand. The cryptic pocket was defined as any residue within 4.5Å of the ligand of the respective co-crystallized complexes. For 6UXY this involved residues 436, 443, 444, 446, 447, 468, 470, 471, 472, 519, 521, 553, 554, 555, 567, 580, 581, 582, 584, 602, and 613. For 6UXX, this included residues 436, 437, 443, 444, 446, 468, 470, 471, 472, 518, 519, 553, 554, 555, 567, 580, 582, 584, 602, and 613.

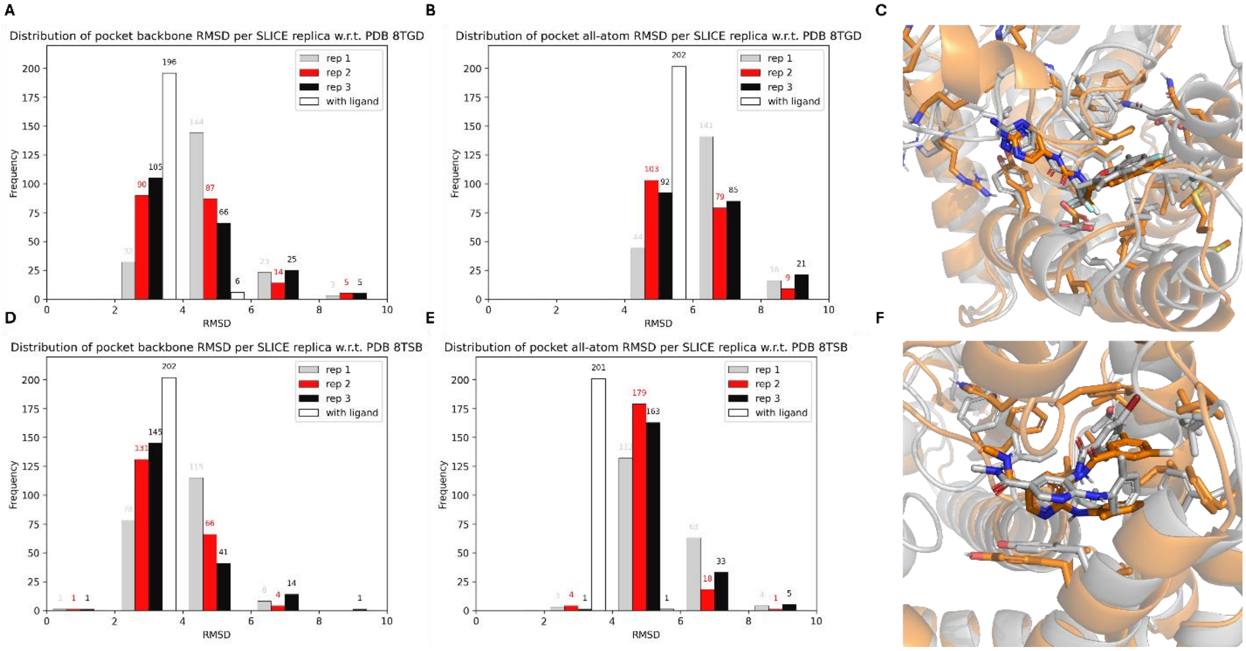

Figure S11. SLICE in the context of the cryptic pocket in PI3Kα. A) Shows the pocket backbone RMSDs per SLICE replica with respect to PDB ID 8TGD. **B**) Depicts the same distribution but considering the all-atom RMSDs. **C**) Illustrates the final snapshot of a 100ns simulation with the cryptic ligand from PDB ID 8TGD (grey) in an open SLICE state (orange). **D**) Shows the pocket backbone RMSD distribution per SLICE replica compared to PDB ID 8TSB. **E**) Shows the same distribution but considering the all-atom RMSDs. **F**) Depicts the final frame of a 100ns simulation with the cryptic ligand from PDB ID 8TSB (grey) in an open SLICE state (orange). The distributions show that SLICE visits backbone states resembling the crystal structure (8TGD or 8TSB) multiple times across replicas. Simulating an open SLICE state with either the 8TGD or 8TSB ligand stabilizes the all-atom RMSD between 2-4Å for both crystal structures. Both ligands also remain stable throughout their 100ns trajectories, as depicted by the overlap with the crystal structures in the last frame of the simulations. The cryptic pocket was defined as any residue within 4.5Å of the ligand in the respective co-crystallized complexes. For 8TGD this includes residues 809, 812, 813, 816, 910, 911, 912, 913, 937, 938, 940, 941, 949, 950, 951, 952, 1002, 1010, 1012, 1013, 1018, 1019, 1021, and 1022. For 8TSB, the considered residue numbers are 809, 812, 813, 911, 912, 913, 937, 941, 1002, 1010, 1012, 1013, 1018, 1019, 1021, and 1022.
